# Effect of Selected Micro- and Macroelements and Vitamins on the Genome Stability of Bovine Embryo Transfer Recipients following In Vitro Fertilization

**DOI:** 10.3390/ani13061056

**Published:** 2023-03-14

**Authors:** Ewa Wójcik, Katarzyna Kępka, Mateusz Skup

**Affiliations:** Institute of Animal Science and Fisheries, Siedlce University of Natural Sciences and Humanities, 14 Prusa St., 08-110 Siedlce, Poland

**Keywords:** Holstein-Friesian, recipient cows, chromosomal instability, minerals, vitamins

## Abstract

**Simple Summary:**

The paper describes a study to assess the stability of the genomes of bovine embryo transfer recipients following in vitro fertilization using cytogenetic tests and to analyze the effects of selected vitamins and micro- and macroelements on genome integrity. Genome stability was analyzed using the sister chromatid exchange, fragile site, and comet assays. The effects of selected micro- and macroelements and vitamins on the levels of chromosomal instabilities generated in the cows were analyzed.

**Abstract:**

Genome instability can lead to a wide variety of diseases. Many endogenous and exogenous factors influence the level of damage to genetic material. Genome integrity depends on factors such as the fidelity of DNA replication, normal DNA organization in the chromosomes, and repair mechanisms. Genome stability influences fertility, embryonic development, and the maintenance of pregnancy. In the case of in vitro fertilization, it can be an important factor determining the success of the procedure. The aim of the study was to assess the stability of the genomes of recipient cows following in vitro fertilization using cytogenetic tests and to analyze the effects of selected vitamins and micro- and macroelements on genome integrity. Genome stability was analyzed using the sister chromatid exchange, fragile site, and comet assays. The material for analysis was peripheral blood from 20 Holstein-Friesian heifers that were embryo transfer recipients. The effect of selected micro- and macroelements and vitamins on the genome stability of the cows was analyzed. Folic acid was shown to significantly influence the level of damage identified using the SCE, FS, and SCGE assays, while iron affected SCE and SCGE results, and zinc affected FS.

## 1. Introduction

Chromosome instability is a form of genome instability indicating increased chromosome damage [1]. Genome integrity is continually subject to disturbances by exogenous and endogenous factors, which can cause various changes in DNA, leading to chromosome damage. Maintenance of genome integrity is crucial in every species for the normal functioning and survival of the organism and the complete transfer of genetic potential to the next generation [2]. Genome stability depends in part on normal DNA organization in the chromosomes, the fidelity of DNA replication, mechanisms responsible for controlling stability, and DNA damage repair pathways. Chromosome instabilities are caused by disturbances in the pre-mitotic phase of the cell cycle, but are also the consequence of the damage and rearrangement of chromosomes resulting from an abnormal cell response to DNA damage or abnormal DNA synthesis [3,4]. Difficulties in analyzing chromosome instability stem from its unclear molecular background. It may result from the effects of non-genetic factors such as physiological processes, e.g., ageing, hormone balance, inflammation, and metabolism, as well as various environmental factors, such as diet.

Vitamins and macro- and microelements help to maintain genome stability and integrity and are an important element of genetic and anti-carcinogenic prevention, because they influence gene expression and the DNA structure [5,6,7]. This is why the quantity and quality of nutrients that influence gene expression and DNA synthesis and repair are so important. Micro- and macroelements and vitamins are cofactors of enzymes catalyzing DNA replication, methylation, and repair [8]. In dairy cattle, they have a major influence on reproduction and productivity [9]. Both deficiencies and surpluses negatively affect balance in the body, disturbing regulatory functions in metabolic processes [10]. Calcium (Ca) is the mineral present in the highest quantities in vertebrates. It is an essential element regulating numerous physiological processes. It activates enzyme activity and has anti-tumor, antiproliferative, proapoptotic, and antimutagenic properties [11]. It takes part in cell division and specialization and in sperm activity, plays an essential role in the fertilization of the ovum, and inhibits chromosome breakage [7,11,12]. Iron (Fe) is another essential microelement for cell function, including the storage and transport of oxygen and numerous enzymes. It supports immune processes in the body and the production of secondary metabolites [13]. It takes part in catabolism and the synthesis of numerous compounds, including nucleic acids; prevents the oxidation of nitrogen bases and the breakage of DNA strands; and regulates the life cycles of cells and the expression of certain genes [14,15]. It is a cofactor of enzymes that break down reactive oxygen species (oxidase, peroxidase, and catalase), protecting cells against oxidative stress, which disturbs the progress of replication and leads to the formation of DNA breaks [16]. Zinc (Zn) influences numerous physiological and metabolic processes and reproduction in animals [9,17]. It takes part in DNA replication, the synthesis of DNA, RNA and proteins, and gene expression [18,19,20]. It is also an essential element for cell proliferation and differentiation, preventing cancer and other diseases [9,20,21,22]. Moreover, it protects the body against the adverse effects of free radicals, preventing DNA lesions and breaks [23]. 

Vitamins are also very important functional substances, which are essential to maintaining health and normal productive, reproductive, and growth parameters, and, in pregnant cows, they prevent embryo death and developmental defects [20]. Vitamins B9 (folic acid) and B12 (cobalamin or cyanocobalamin) prevent DNA strand breaks and the oxidation of bases and are involved in the synthesis of nitrogen bases and in DNA transcription and repair. Anomalies in these biological functions result in an increased number of lesions in genetic material and a reduction in DNA methylation, which can lead to developmental defects and carcinogenesis. Folic acid has a strong influence on genome stability. A deficiency of folic acid in the diet leads to errors in DNA synthesis, changes in the degree of methylation, and chromosome breaks. This is why a suitably balanced diet for pregnant cows is so important [24,25,26,27,28]. Sites in chromosomes with CGG repeats are particularly sensitive to folic acid deficiencies, which can lead to the defective segregation of chromosomes in these sequences and even cause instability of the entire chromosome [29,30]. A B9 deficiency leads to a number of disorders of the nervous and digestive systems, inhibition of cell growth and regeneration, reduced milk yields in cows, chromosomal disorders, the transfer of abnormal amounts of DNA, and fetal defects [25,26,27,28]. Vitamin B12 is closely correlated with folic acid [31]. It plays an important role in cell growth and development by participating in numerous reactions and processes in the body. In combination with folates, it influences hematopoietic processes, DNA synthesis, and the methylation of DNA and RNA. It also has anti-tumor effects and additionally takes part in erythropoiesis; the metabolism of carbohydrates, fats, and nucleic acids; and neuron function, regulating mental processes [32,33]. Vitamin B12 deficiency leads to neurological disorders, apathy, loss of appetite, anemia, reduced body condition and productivity, and DNA damage [34]. Excessive intake of concentrate feed increases the concentration of propionic acid in the rumen, which prevents the production of adequate amounts of cobalamin. 

Micro- and macroelements and vitamins are important regulators of DNA synthesis and repair. This is why a suitably balanced diet with the most important minerals and vitamins ensuring genome stability is so important. Cytogenetic tests are sensitive methods for detecting chromosome damage, providing information about genome stability. They include the sister chromatid exchange assay, fragile site assay, and comet assay. They also provide information about the potential for the repair of DNA damage, the control of the entire cell cycle, and the effects of malfunctioning cellular mechanisms responsible for maintaining stability. The sister chromatid exchange assay is used to quantify genetic material exchanged between sister chromatids during mitosis. The test makes it possible to detect DNA damage in the form of single- and double-strand breaks induced by mutagenic and genotoxic factors [35]. 

A sister chromatid exchange (SCE) is associated with malfunctioning repair pathways [36], leading to the fragmentation of sister chromatids as a result of DNA strand breakage and resealing. This rearrangement is accompanied by the exchange of regions of the parent strands in the duplicated chromosomes between sister chromatids, which is possible owing to cohesion between chromatids [37,38]. Fragile sites (FS) in chromosomes are sites that are especially sensitive to breaks, gaps, and constrictions under replication stress [39]. They are the consequence of malfunctioning mechanisms of repair of disturbances in the progression of replication forks during replication and transcription [36]. The lack of complete replication and repair of DNA damage at FS results in the transfer of genome damage and instability between generations of cells [39]. FS are regarded as hotspots of chromosome instability and rearrangement in cancer [24]. Depending on their frequency in the population and means of induction, rare fragile sites (RFS) and common fragile sites (CFS) are distinguished [40,41]. The former, which occur in up to 5% of the population, are inherited according to the laws of Mendel and are classified based on their sensitivity to a lack of folic acid (folate-sensitive vs. non-folate-sensitive) [41,42,43]. They most often take the form of numerous trinucleotide repeats, or, less often, di- or tetranucleotide repeats. For folate-sensitive fragile sites, they are CCG trinucleotide repeats, while, in the case of non-folate-sensitive FS, they are AT-rich repeated sequences. The high elasticity of repeated sequences affects the dynamics of replication, reduces the effectiveness of links between nucleosomes, and leads to the decondensation of genetic material [41,43]. Repeated nucleotide sequences are able to form secondary and tetrahelical structures that block replication forks, resulting in delayed replication [42,43,44]. CFSs are an integral element of the chromosome structure. They occur in the genome spontaneously and affect large genomic regions consisting of hundreds or even thousands of kilobases [40,45,46]. They include AT-rich nucleotide sequences without repeats, do not show a tendency to expand, and are arranged in the form of islands, which increases the elasticity of the sequences [42,47]. As in the case of RFSs, in CFSs, secondary structures also cause disturbances in replication and higher-order chromatin organization [43,46]. 

The comet assay, i.e., single-cell gel electrophoresis (SCGE), is a rapid and sensitive technique for the identification of DNA degradation in individual cells. An advantage of this method is that it can detect various types of errors, e.g., DNA single- and double-strand breaks, alkali-labile sites, DNA single-strand breaks associated with incomplete excision repair sites, and apurinic/apyrimidinic sites. The comet assay therefore identifies many types of DNA damage leading to instability and mutations induced by genotoxic and mutagenic stress factors, such as ionizing radiation [48,49,50,51,52]. A major advantage of this test is that a great many individual cells can be analyzed, which enables a more precise analysis of instabilities in the genetic material [53].

Eating disorders deregulate genetic, epigenetic, and epigenomic mechanisms. Improper intake of minerals, vitamins, and, importantly, folates is correlated with abnormalities in fetal programming, an increased risk of complications during fetal development, and the occurrence of diseases in adult life [54]. Disproportions in nutrients and deficiencies of vitamins and minerals can lead to functional disorders in the adult animal and developmental disorders in the fetus obtained from in vitro fertilization. Assisted reproductive techniques are used not only in cases of infertility, but also to increase reproductive performance and accelerate breeding progress. This is particularly important in cattle, due to the relatively long generational interval in comparison with other livestock species. Therefore, in vitro fertilization is a very important issue in breeding. It makes it possible to produce embryos of high genetic value, combat reproductive disorders, and predict fertility. The OPU/IVP (ovum pick-up/in vitro production) method consists of numerous stages: the acquisition of male and female gametes, bringing them to fertilization capacity; the fusion of the oocyte with the spermatozoon via IVF (in vitro fertilization) or microinjection of the spermatozoon into the cytoplasm of the oocyte; the in vitro culture of the embryo; the evaluation of the embryo up to the morula and blastocyst form; and the transfer of the embryos into the recipient [55,56]. An increasing number of breeders are using modern reproductive techniques in their herds (e.g., sexed semen, embryo transfer, or in vitro fertilization). For the process to be successful, each step is controlled regarding the transfer and deposition of the embryos in the reproductive tract of the cow [56].

Genetic and nutritional factors have a major impact on processes. In recipients, these factors affect the maintenance of the pregnancy and the unassisted birth of healthy calves [57]. The choice of recipients with a stable genome and optimal levels of micro- and macroelements and vitamins in the body vastly improves the chance of obtaining healthy calves and future adult animals [10,58]. The analysis of genome stability using cytogenetic techniques and the assessment of levels of micro- and macroelements and B vitamins makes it possible to monitor the health potential of recipient cows and their calves, and thus the cows’ diet is an important element in the preparation of cows for fertilization. 

The aim of the study was to assess the genome stability of bovine embryo transfer recipients following in vitro fertilization using cytogenetic tests and to analyze the influence of selected vitamins and micro- and macroelements on genome integrity.

## 2. Materials and Methods

### 2.1. Animals

All experiments were conducted in accordance with the recommendations in Directive 63/2010/EU and the Journal of Laws of the Republic of Poland of 2015 on the protection of animals used for scientific or educational purposes. The study was approved by the Polish Laboratory Animal Science Association (nos. 3235/2015 and 4466/2017).

The research material was peripheral blood drawn from the tail veins of 20 Holstein-Friesian cows at the age of 18–20 months. The blood was obtained from bovine embryo transfer recipients following in vitro fertilization, in roughly the third month of gestation. All cows were from the same herd.

### 2.2. Cell Culture 

Peripheral blood lymphocytes were cultured in vitro in Lymphogrow growth medium for 72 h at 38.5 °C (5% CO, with stable humidity). At 69 h of culture, colchicine was added (2.5 µg mL^−1^). At 24 h, 5-bromodeoxyuridine (BrdU) was added to the cultures intended for SCE assays (10 µg mL^−1^), and at 65 h, BrdU was added to the cultures for the FS test (5 µg mL^−1^). Potassium chloride (0.65% KCl) was used as a hypotonic solution. The cells were fixed with Carnoy fixative (3:1 methanol–acetic acid).

### 2.3. Sister Chromatid Exchange Assay

The FPG technique [59] was used to detect sister chromatid exchanges in the following steps: digestion with 0.01% RNase, incubation in a solution of 0.5 × SSC (sodium chloride + sodium citrate; pH = 7.0) with Hoechst 33258 solution, UV irradiation twice, overnight incubation at 4 °C, incubation at 58 °C, and 4% Giemsa staining. Stained sister chromatid exchanges were counted in 20 metaphases from each individual.

### 2.4. Fragile Site Assay

Microscope slides for the identification of fragile sites were prepared using the technique of differential staining chromosomes in the following steps: incubation in Hoechst 33258 solution (1 mg/100 mL), UV irradiation, incubation in 2 × SSC, and 4% Giemsa staining. Twenty metaphases were examined from each individual. Chromatid breaks, chromatid gaps, and chromosome breaks were identified.

### 2.5. Comet Assay

The single-cell gel electrophoresis (SCGE) assay (comet assay) was performed on microscope slides [60]. Lymphocytes were isolated with Histopaque-1077. Slides coated with a layer of 0.5% normal melting point (NMP) agarose gel were spotted with lymphocytes mixed with 0.5% low melting point (LMP) agarose gel and then embedded in LMP agarose. Samples prepared in this manner were subjected to alkaline lysis (2.5 M NaCl, 100 mM Na_2_EDTA, 0.4 M Tris-HCl, 1% sodium N-lauroylsarcosinate, 10% Triton X-100, 1% DMSO, pH = 10) to release DNA from the cell and remove proteins. This was followed by alkaline denaturation in electrophoresis solution, neutralization with Tris-HCl, and staining with ethidium bromide. DNA integrity was determined on the basis of the percentage content of DNA in the tail (%T DNA) of the comet. Fifty cells were analyzed for each animal. Changes observed in cells were classified according to Gedik’s scale: N—no DNA damage or less than 5% damage in the comet tail; L—low level of damage (5–25%); M—moderate damage (25–40%); H—high level of damage (40–95%), and T—over 95% DNA damage [61].

### 2.6. Analysis 

An Olympus BX50 microscope was used for microscopic analysis. The MultiScan image analysis software from Computer Scanning Systems was used to analyze chromosome damage identified in the form of sister chromatid exchanges and fragile sites. The CASP 1.2.2 software [62] was used to analyze degraded DNA of lymphocytes identified by the comet assay. 

Levels of micro- and macroelements (Ca, Fe, and Zn) and vitamins (B9 and B12) in the blood serum were tested by a commercial veterinary laboratory. The results were presented in a table. The reference values for the minerals and vitamins were Ca 2.3–2.8 mmol/L; Fe 25–35 µmol/L; Zn 10.7–19.9 µmol/L; B9 17–24 ng/mL; B12 150–200 pg/mL [63].

Statistical analysis of the results was performed using STATISTICA 12.5 MR1 PL software. The effect of the level of vitamins (B9 and B12) and minerals (Ca, Fe, and Zn) on the frequency of chromosomal instabilities (SCE, FS, and SCGE) was assessed. Means between individuals within groups were compared by one-way ANOVA. Significance of differences between means for a given type of instability within factors was assessed by Tukey’s test (*p* < 0.05). In addition, the relationships between the content of vitamins and minerals and the level of damage were tested by correlation analysis. Simple regression equations were built for significant relationships. All calculations were performed for *p* < 0.05.

## 3. Results

**Figure 1:**
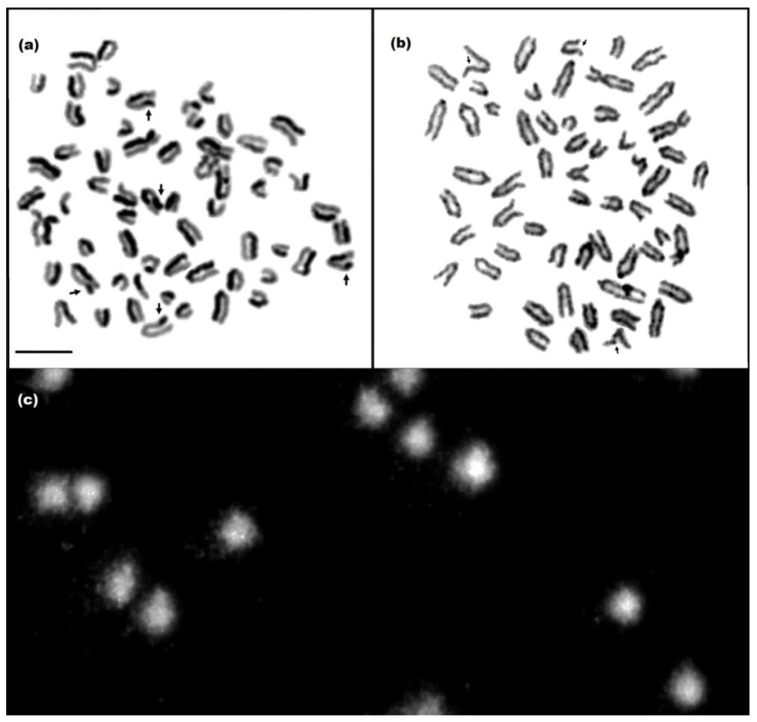
Mitotic chromosomes in the metaphase stained by the SCE assay (**a**) and FS assay (**b**), and cell nuclei of lymphocytes subjected to comet assay (**c**). Damage marked with arrows. Scale bar 10 µm.

The study evaluated the genome stability of cows following in vitro fertilization. Figure 1 present images of metaphase chromosomes tested by the SCE and FS assays and nuclei of lymphocytes tested by the comet assay. In addition, the blood serum of the cows was analyzed for the content of Ca, Fe, Zn, and vitamins B9 and B12, and the effect of the micro- and macroelements on the integrity of the genetic material was assessed. 

The average frequency of SCEs in the cows was 5.0 ± 7.9 SCEs/cell. Differences were shown between cows in the average frequency of SCEs, including statistically significant differences between the cows with numbers 6, 7, and 17 and those with numbers 2, 3, 8, 9, 11, and 16. The most SCEs were noted in cow no. 16, and the fewest in no. 17 (Table 1). The average frequency of FS was 3.2 ± 1.1 FS/cell. Differences in FS frequency were shown between cows, but were statistically significant only between no. 16 and all other cows. The frequency of this type of damage was highest in cow no. 16 and lowest in nos. 1 and 17 (Table 1). The average %T DNA in the cows was 3.8 ± 1.4. Differences were found in the frequency of damage identified by the comet test, including statistically significant differences, e.g., between no. 1 and nos. 3, 11, and 16. The most damage was observed in cow no. 16, and the least in no. 17 (Table 1). The low frequency of damage to both DNA and chromosomes in the cattle suggests that the animals’ genome was stable. Based on an additional criterion, i.e., Gedik’s scale, the animals were classified as N (14 cows) or L (6 cows), with a low level of DNA damage. N indicates no DNA damage or less than 5% damage in the comet tail, while L represents a low level of damage (5–25%).

Table 2 presents analyses of minerals and vitamins. Serum levels of Ca in all cows were within the reference range, averaging 2.5 mmol/L. The average serum concentration of Fe was 34.8 µmol/L. The Fe level was elevated in eight cows (nos. 2, 4, 8, 9, 10, 11, 13, and 20), but within the reference range in the others. The average Zn level was 14 µmol/L. The cows with nos. 11, 13, 16, and 18 had low Zn values, below the reference range, while cows 1, 3, and 5 had elevated values. The average content of vitamin B9 in the blood of the cows was 12.4 ng/mL. Vitamin B9 levels were outside the reference range in most of the individuals; only cow no. 17 had a correct concentration. Four cows (nos. 1, 2, 6, and 7) had elevated levels of vitamin B9, while its level was low in the others. The average vitamin B12 level in the blood of the cows was 151.1 pg/mL. Only cows no. 8 and 9 had correct concentrations of this vitamin, while, in all the others, it was low. 

Analysis of variance showed that the number of SCE, FS, and SCGE instabilities depends on the concentration of vitamin B9 in the blood. The highest levels of SCE, FS, and SCGE instability were noted when the B9 level was very low, while the least damage was detected when its content was within reference values. Analysis of the correlation between the vitamin B9 concentration and the number of lesions showed a significant negative correlation (Table 3). As the vitamin B9 concentration in the blood increased, the number of instabilities decreased. A 1 ng/mL increase in the vitamin B9 level caused a decrease in the level of damage by 0.06 for SCE, 0.038 for FS, and 0.206% for SCGE (Table 4).

The iron level in the blood did not significantly influence the level of SCE and FS instability, but significantly influenced the level of damage detected by SCGE. The number of lesions detected in SCGE was highest when the iron level was higher than the reference values and lowest when it was below the reference values. Analysis of the correlations between the iron content in the blood and the level of instability showed a positive correlation between the concentration of this microelement and SCE and SCGE damage (Table 3). A 1 µmol/L increase in the iron level was associated with a 0.046 increase in SCEs and a 0.21% increase in the SCGE result (Table 4). 

In the case of zinc, analysis of variance showed a significant relationship between the number of FS and the content of this element in the blood. Deviations from the reference values (above or below) were associated with an increase in the level of chromosome damage. Zinc levels in the blood did not significantly influence the number of SCE and SCGE instabilities. Analysis of the correlation between the zinc level in the blood and the level of damage to genetic material showed no significant relationships (Table 3). Analysis of variance and correlations showed no significant relationships between concentrations of vitamin B12 and calcium in the blood and the level of SCE, FS, and SCGE damage. 

## 4. Discussion

Cattle are the most widespread species of livestock. Intensive evaluation of the use value and breeding value has resulted in a vast increase in milk performance. Selection for high yields has unfortunately been carried out at the cost of health and reproductive parameters [64]. A milestone in the process of improvement of cattle was the development of biotechnological breeding methods (e.g., cryopreservation of semen, artificial insemination, in vitro fertilization, and embryo transfer), resulting in healthier animals with high milk yields [65]. A helpful tool for achieving this goal is in vitro fertilization, which makes it possible to select donor cows with the desired traits [57,66,67]. Maintenance of pregnancy and the birth of a healthy calf are associated not only with the recipient cow’s physical condition, but also determined by the integrity of the genetic material. Studies assessing genome stability in cows used in assisted reproduction, e.g., in vitro fertilization, can make it possible to evaluate and select the best cows, i.e., those with high genome stability. Studies analyzing chromosomal instabilities in cattle most often focus on numerical and structural mutations in chromosomes [3,68,69,70]. There are fewer studies on chromosomal instabilities resulting from errors in replication, transcription, or malfunctioning repair mechanisms and control points tasked with catching these errors. They can be identified using cytogenetic tests, such as the sister chromatid exchange, fragile site, and comet assays. These are extremely valuable tools for evaluating animals, because they are highly sensitive and provide information about malfunctions in a number of important cellular processes responsible for maintaining genome stability and integrity [71]. 

According to Danielak-Czech and Słota [72], Nino-Soto and King [73], and Danielak-Czech et al. [74], instabilities negatively affect reproduction in cattle by extending the calving interval, reducing the effectiveness of artificial insemination, or causing the loss of embryos. Luna et al. [75] also found that a high number of chromatid breaks and gaps causes reproductive problems in cows. Many mutagenic, genotoxic, and carcinogenic factors negatively affect animal health [76,77]. Abnormalities occurring during cell division can generate various forms of damage to genetic material, resulting in abnormally developed embryos, which die in the early stages of embryonic development [78,79]. Abortion results in new, delayed estrus, prolonging the calving interval and thus generating further economic losses for the breeder [69]. The group of cows analyzed in the present study was homogeneous in terms of age, breed, and location. According to researchers, these factors significantly influence the frequency of instabilities [36,80,81,82,83,84,85]. Therefore, the results obtained are a reliable indicator of the level of genome stability in the recipient cows, following the exclusion of the above-mentioned factors that negatively affect genome integrity. The level of damage observed in our study was low (SCE 5.0, FS 3.2, and %T DNA 3.8). According to Azimi [86], the average frequency of SCEs for healthy cattle ranges from 5 to 14. Deviations from this standard indicate pathological changes in the body [87]. According to Di Meo et al. [88,89], Peretti et al. [90], and Wójcik et al. [91], the incidence of SCEs is characterized by species conservatism. Our analysis of various studies revealed that the frequency of spontaneous SCEs in cows ranges from 3.2 to 8.3 [80,82,85,86,92]. This wide range of averages is influenced by the breed of cow. Lower SCE values have been observed in indigenous cow populations, while the frequency of these instabilities in Holstein-Friesian cows was 5.1, 6.8, 7.1, 7.1, and 8.3 [80,85,86]. The mean SCE values obtained in our study are lower than in those cited above, which indicates high genome stability in the recipient cows. Unfortunately, there are no published reports of the results of the identification of SCE, FS, and DNA analyzed by the comet test in cows impregnated following in vitro fertilization. Fragile sites have been identified in cattle chromosomes by Peretti et al. [71], Wójcik and Szostek [85], Danielak-Czech and Słota [93], Di Meo et al. [94], and Genualdo et al. [95]. The frequency of spontaneous FS ranged from 0.21 to 3.0/cell. The average FS frequency of 3.2 obtained in our study is similar to the results reported by Wójcik and Szostek [85] (3.5), Danielak-Czech and Słota [93] (3.0), and Rodriguez et al. [96] (2.5). There is also very little information on the use of the comet test in cattle. It has been used in genetic toxicology monitoring, e.g., of the effect of ivermectin, copper, or lead on the DNA structure [97,98,99,100], and to identify damage to genetic material induced by oxidative stress in cow embryos [101] and in cows with Bovine papillomavirus [102]. Tharwat et al. [103] also used SCGE to investigate apoptosis in the peripheral blood cells of dairy cows three weeks before expected parturition, during the week of parturition, and after three weeks. They observed a higher frequency of DNA damage in the week of parturition and three weeks after parturition than during pregnancy. According to the authors, these findings are explained by endocrine changes, immunosuppression in the peripartum period, changes in diet, metabolic and immune disorders, and stress factors associated with the change of location in the cowshed.

Genome stability is determined by many traits, as well as by exogenous factors such as diet [84]. Pregnant cows should have a suitably balanced diet, which is a key factor influencing the levels of micro- and macroelements and vitamins in cattle. Supplementation reduces the risk of stillbirths and disease in the mothers [104]. Failure to monitor the composition of the feed and blood biochemical parameters when correcting deficiencies, or the use of feed additives that are not adjusted to the animals’ needs, can disturb homeostasis and may also cause toxicosis during pregnancy, leading to abortion or stillbirths [10,105]. Deficiencies of vitamins and minerals, especially Zn, are especially dangerous. Embryo transfer to heifers with inadequate concentrations of these elements, or of total protein, urea nitrogen, albumins, and beta-carotene, accompanied by an elevated total bilirubin concentration and excessive nitrogen intake, is associated with problems with embryonic development and implantation. Disorders of fat metabolism reduce the secretion of progesterone, which is responsible for maintaining pregnancy [105]. The major organs in the fetus are formed in the first few months of pregnancy. This is why it is so important to provide the mother with a suitably balanced diet, to avoid deficiencies that could adversely affect the stability of the genetic material of the mother and fetus [105,106,107]. Minerals such as calcium, iron, and zinc are important elements influencing physiological functions, genetic resistance, and reproductive functions [108]. In the present study, all cows had normal serum levels of calcium. A correct Ca level has a positive effect on genetic resistance and productivity, but its quantity is also correlated with the bioavailability of zinc [11,109]. In our study, four recipient cows had low Zn levels, and much higher frequencies of SCEs were shown in these cows in comparison to the others. According to Gressley [9], Seyrek et al. [18], and Omur et al. [20], Zn is an essential element preventing oxidative stress, which adversely affects the progress of replication forks and causes DNA single- and double-strand breaks. The SCE assay proved to be a highly sensitive diagnostic tool in detecting the negative effect of Zn deficiency on the cows’ genetic material. According to Mirowski [19] and Meglia et al. [21], Zn deficiencies can be a consequence of increased stress but also of an unbalanced diet and colostrum production. Excess zinc can lead to tumor formation and reduce the bioavailability of calcium, iron, copper, and phosphorus [110]. In our study, cows with elevated Zn had slightly lower levels of Ca and Fe than other cows, but the values were within the reference ranges. No negative effect of the elevated Zn level on the genetic material was observed. Some cows had elevated Fe levels in the blood. As the Fe level increased, the frequency of damage detected by the SCE and comet assays increased as well. Sanders et al. [100] also used the comet assay and other molecular techniques to determine that excess Fe contributes to oxidative stress, cytotoxicity, and genotoxicity in cells and identified an increase in DNA damage. A low level of Fe, according to Regmi and Dhakal [108], causes reproductive failure, e.g., embryonic death.

Group B vitamins are crucial to preserving genome stability. In the present study, the level of vitamin B9 was normal in only one cow; it was elevated in four and low in the others. In the case of B12 as well, only two cows had normal concentrations of this vitamin, while in the others, it was below the norm. Folic acid is an essential nutrient that plays a crucial role in immune processes and prevents disease in pregnant animals. In the present study, as the vitamin B9 concentration increased, the frequency of chromosomal instabilities identified by the SCE, FS, and comet assays decreased. Folic acid plays an important role in the expression of genes, including those responsible for immunity [28]. It takes part in DNA synthesis, transcription, and repair. The consequences of disturbances in these biological functions include an increased frequency of damage to genetic material and a reduced level of DNA methylation [7,31]. Cobalamin is closely associated with folic acid. Girard and Matte [31,111] reported that cows with low serum levels of B12 did not react positively to supplementation with folates. According to Mirowski [112], low levels of this vitamin adversely affect reproduction, metabolism, and health status in cows. Khan et al. [27] also claim that folic acid and vitamin B12 are essential nutrients that influence the level of metabolic and immune stress during pregnancy and the peripartum period. According to Mirowski [19] and Kincaid and Socha [113], levels of vitamins and minerals in advanced pregnancy are varied, e.g., the level of zinc in the blood falls as pregnancy advances, possibly due to impending parturition and lactation. Therefore, it is essential to monitor micro- and macroelements and vitamins in pregnant cows at various stages of advancement of pregnancy.

## 5. Conclusions

The analyses showed which of the vitamins and minerals affect the stability of genetic material. The level of chromosome instability detected by the SCE, FS, and SCGE assays significantly depends on the level of vitamin B9 in the blood. As its content increases, the level of damage decreases. SCE and SCGE instability also depends on the iron level in the blood. The number of FS significantly depends on the zinc concentration in the blood; deviations from reference values of zinc cause an increase in the level of this type of damage. Higher content of this element increases the number of these lesions. Zinc content did not significantly increase the number of SCE and SCGE instabilities. No relationship was found between the level of SCE, FS, or SCGE chromosome instabilities and the concentration of calcium or vitamin B12 in the blood.

## Figures and Tables

**Table 1:** Number of instabilities identified (SCE, FS, and SCGE) in cows. Means designated with different letters vary significantly at *p* < 0.05.

Cow	SCE	FS	SCGE
	Mean ± SD		
1.	4.6 ^ab^ ± 1.1	2.8 ^a^ ± 0.7	2.1 ^ab^ ± 4.6
2.	5.4 ^bc^ ± 1.1	3.5 ^ab^ ± 1.1	5.7 ^bcd^ ± 11.9
3.	5.5 ^bc^ ± 1.4	3.3 ^ab^ ± 0.8	7.1 ^cd^ ± 11.8
4.	5.3 ^abc^ ± 1.3	3.4 ^ab^ ± 0.9	4.7 ^abcd^ ± 10.9
5.	5.1 ^abc^ ± 1.4	3.3 ^ab^ ± 0.9	3.6 ^abcd^ ± 8.3
6.	4.1 ^a^ ± 1.3	2.6 ^a^ ± 0.9	0.8 ^a^ ± 2.8
7.	4.0 ^a^ ± 1.2	2.5 ^a^ ± 1.0	0.6 ^a^ ± 2.3
8.	5.4 ^bc^ ± 1.5	3.3 ^ab^ ± 1.0	5.3 ^abcd^ ± 10.5
9.	5.4 ^bc^ ± 1.2	3.4 ^ab^ ± 1.2	6.7 ^bcd^ ± 8.4
10.	5.2 ^abc^ ± 1.3	3.0 ^ab^ ± 0.7	4.6 ^abcd^ ± 10.9
11.	5.8 ^bc^ ± 1.5	4.0 ^bc^ ± 1.3	8.3 ^cd^ ± 6.7
12.	4.6 ^ab^ ± 1.1	3.0 ^ab^ ± 0.8	1.7 ^abc^ ± 4.7
13.	5.0 ^abc^ ± 1.3	3.2 ^ab^ ± 1.0	3.3 ^abcd^ ± 7.6
14.	4.4 ^ab^ ± 1.3	3.0 ^ab^ ± 1.0	1.7 ^ab^ ± 4.9
15.	4.7 ^ab^ ± 1.3	2.9 ^a^ ± 0.9	2.1 ^ab^ ± 6.4
16.	6.4 ^c^ ± 1.1	4.8 ^c^ ± 0.8	9.1 ^d^ ± 12.0
17.	3.9 ^a^ ± 1.3	2.8 ^a^ ± 0.8	0.5 ^a^ ± 2.5
18.	4.7 ^ab^ ± 1.4	3.1 ^ab^ ± 1.1	2.9 ^abcd^ ± 6.2
19.	4.8 ^ab^ ± 1.5	3.2 ^ab^ ± 1.1	2.4 ^ab^ ± 6.0
20.	5.1 ^abc^ ± 1.2	3.2 ^ab^ ± 0.8	3.3 ^abcd^ ± 9.3

^abcd^ mean values with different superscript letters are statistically different (*p* < 0.05) between breeds.

**Table 2:** Content of selected micro- and macroelements and vitamins in the blood serum of cows.

Cow	Ca	Fe	Zn	B_9_	B_12_
	[mmol/L]	[µmol/L]	[µmol/L]	[ng/mL]	[pg/mL]
1.	2.3	29.2	>26.5	>24.0	<150
2.	2.5	>45.9	18.7	>24.0	<150
3.	2.4	31.6	>24.4	<5.3	<150
4.	2.5	>40.1	14.6	<9.4	<150
5.	2.4	29.7	>22.7	<6.5	<150
6.	2.5	33.2	13.3	>24.0	<150
7.	2.5	33.4	11.1	>24.0	<150
8.	2.5	>36.1	16.6	<12.2	160
9.	2.4	>45.9	11.8	<6.1	161
10.	2.4	>40.7	12.3	<10.6	<150
11.	2.4	>44.7	<9.9	<6.2	<150
12.	2.6	28.3	12.0	<12.0	<150
13.	2.4	>42.5	<9.2	<9.9	<150
14.	2.6	28.2	10.3	<12.2	<150
15.	2.5	29.8	11.8	<8.1	<150
16.	2.5	31.5	<9.3	<5.6	<150
17.	2.5	28.9	12.4	19.4	<150
18.	2.5	30.3	<10.6	<7.0	<150
19.	2.5	28.1	11.1	<14.9	<150
20.	2.5	>37.7	12.3	<6.2	<150

**Table 3:** Correlations between levels of damage and the content of selected minerals and vitamins in the blood.

Test	B_9_	Ca	Fe	Zn
SCE	−0.612732	−0.020744	0.476745	0.191529
FS	−0.530984	0.089813	0.305435	−0.025100
SCGE	−0.560572	−0.035294	0.534740	0.227794

**Table 4:** Regression equations describing the relationship between levels of damage and the content of vitamin B9 and Fe.

Test	B_9_	Fe
SCE	y = 5.65 − 0.06x (*p* = 0.00)	y = 3.34 + 0.046x (*p* = 0.03)
FS	y = 3.69 − 0.038x (*p* = 0.011)	
SCGE	y = 6.38 − 0.206x (*p* = 0.010)	y = −3.59 + 0.21x (*p* = 0.015)

## Data Availability

This study did not report any data.
